# The Hydroarylation Reaction—Scope and Limitations

**DOI:** 10.3390/molecules15053402

**Published:** 2010-05-10

**Authors:** Jan C. Namyslo, Jörg Storsberg, Jens Klinge, Christian Gärtner, Min-Liang Yao, Nuket Ocal, Dieter Eckhard Kaufmann

**Affiliations:** 1 Institute of Organic Chemistry, Clausthal University of Technology, Leibnizstr. 6, D-38678 Clausthal-Zellerfeld, Germany; 2 Department of Chemistry, Faculty of Arts and Sciences, Davutpasa Campus, Yildiz Technical University, 34210 Esenler-Istanbul, Turkey

**Keywords:** homogenous catalysis, palladium, C-C coupling, domino reaction, rearrangement, mechanism

## Abstract

The synthetic potential of stereoselective, palladium-catalyzed hydro(het)arylation reactions of bi-, tri- and tetracyclic (hetero)alkenes in the presence of phospines and arsines as highly efficient ligands was studied. The mechanism of this reductive Heck reaction becomes more complex in the case of benzonorbornenes. Hydroarylation of diazabicyclo-[2.2.1]heptenes provides a stereoselective access to aryldiaminocyclopentanes. Electron-deficient arylpalladium complexes shift the reaction towards the product of a formal 1,2-hydrazidoarylation reaction of 1,3-cyclopentadiene by a stereoselective C-N cleavage. Due to steric reasons, rigid bicyclo[2.2.2]octenes react slower in hydroarylation reactions than the corresponding bicyclo[2.2.1]heptenes. The more flexible bicyclo[4.2.2]decene system already tends to undergo domino-Heck reactions, even under reductive conditions. When a tetracyclic *cis*-allylcyclopropane is carbopalladated in the presence of formates, the neighboring cyclopropane ring is attacked in the first reported example of a π,σ domino-Heck reaction.

## 1. Introduction

The development of new methodologies for stereoselective C-C coupling reactions in order to synthesize complex structures from easily available precursors has attracted much interest in contemporary synthetic chemistry. Among them, the transition metal-catalyzed reactions, especially the *Heck* and related reactions have become more and more important during the last two decades [1,2,3,4,5,6,7,8]. This palladium-catalyzed arylation provides high yields with open-chain and cyclic alkenes. In the case of rigid bicyclic alkenes a reductive variant appears feasible. The *Catellani* reaction uses norbornene as a template for *ortho*-substitution [9]. Since the first catalyzed reductive phenylation of norbornene was published in 1989 [10,11] the great synthetic potential of the hydroarylation reaction with bicyclic alkenes, including its asymmetric variant with bicyclic alkenes for the one-step construction of up to four asymmetric centers has prompted us to further investigations [12,13,14,15,16].

## 2. Results and Discussion

**Scheme 1:**
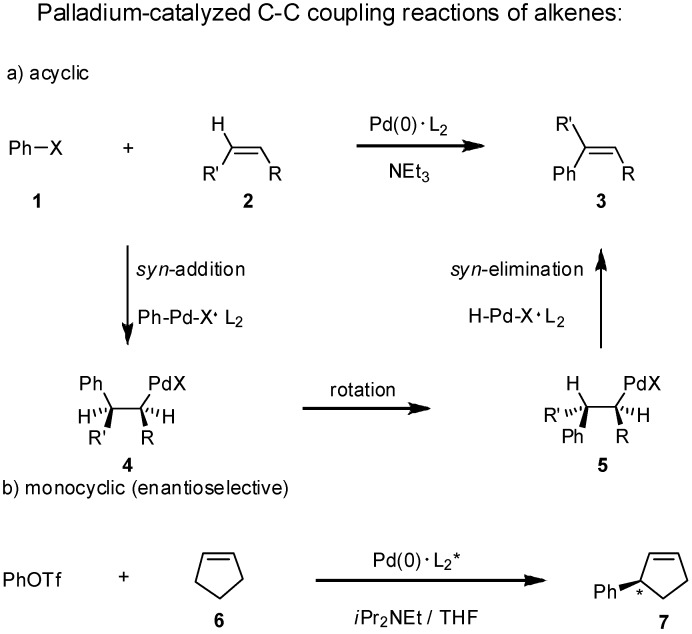
The Heck reaction of acyclic and monocyclic alkenes **2** and **6**.

Starting from an open-chain alkene **2** and a halogenated arene **1** three reaction steps lead to the stereoselective formation of a styrene derivative in the presence of a stabilizing ligand L. The first step is a *syn*-carbopalladation of the alkene giving **4**, followed by rotation around the central single bond in **5** to allow the final step, the *syn*-dehydropalladation giving a styrene derivative **3**. In the presence of chiral ligands L_2_*, such as sugar-based, chiral phosphite-oxazolines the Heck reaction of monocyclic alkenes like cyclopentene (**6**) proceeds with high regio- and enantioselectivity generating one asymmetric center [17] (Scheme 1).

**Scheme 2:**
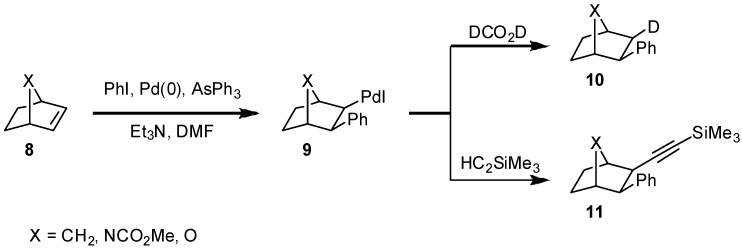
Intermolecular consecutive reactions of a carbopalladation product **9** from **8**.

Rigid bicyclic alkenes **8** cannot be arylated this way as the final *syn*-dehydropalladation of intermediate **9** is not feasible due to geometrical constraints; inter- or intramolecular consecutive reactions of the carbopalladation product **9** such as reduction or alkynylation (**10**, **11**) work well [7]. Many applications for the reductive variant of the Heck reaction are known and the reductive arylation of bicyclic alkenes such as norbornene and its aza- or oxa-analogues **8** using palladium catalysts has been well studied [18] (Scheme 2).

Both, the carbopalladation to yield **9** and the consecutive substitution reactions proceed with complete *exo*-diastereoselectivity. This was a very important finding for the stereoselective synthesis of the highly bioactive alkaloid epibatidine [19], the parent amine of **13**. Epibatidine, which is a lead in the development of new analgesics, is accessible by hydroarylation of the 7-azabicyclo-[2.2.1]heptene system with 6-chloro-3-iodopyridine. In the presence of chiral ligands, three asymmetric centers can be controlled. The epibatidine enantiomers show different biological activities. For biological testing, analogues modified in the 7-position such as **12**, **14** have been synthesized. The enantiomeric excesses achieved are strongly dependent on the ligands employed. The P,P-ligand (*R*)-BINAP (**15**) is well suited for the synthesis of the epibatidine derivative **13** (81% e.e.), whereas the P,N-ligand (*S*)-Ms-Valphos **16** proved to be the best ligand for the hydrohetarylation of the carbon analogue norbornene. Both ligands give low enantiomeric excesses in case of the oxa analogue, though (Table 1).

**Table 1:** Asymmetric hydro(hetero)arylation of bicyclo[2.2.1]heptene and its 7-hetero- analogues: e.e. (c.y.) [%] of the products **12**-**14**.

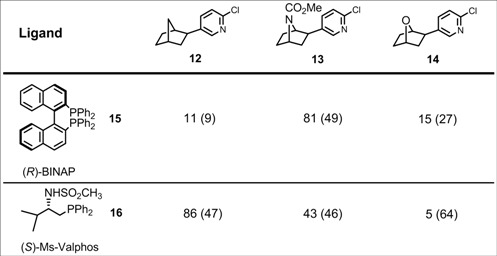

**Scheme 3:**
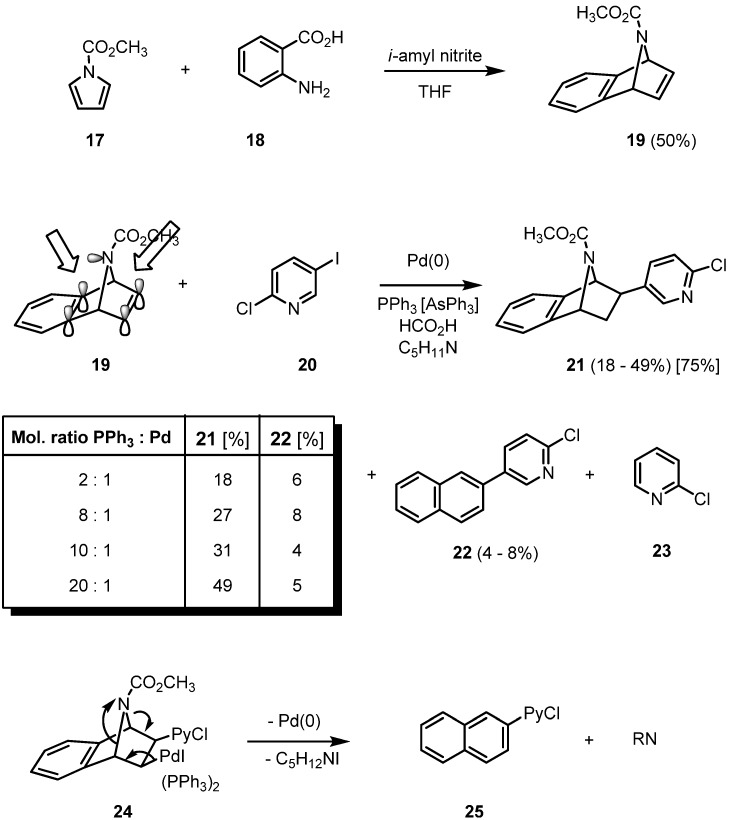
Reductive Heck arylation of **19** with 2-chloro-5-iodopyridine (IPyCl, **20**).

The reductive Heck arylation can be extended to the more lipophilic benzoannelated epibatidine analogues. They are easily accessible by a Diels-Alder reaction of cyclopentadiene, furan or *N*-methoxycarbonylpyrrole (**17**) with benzyne, generated from the diazotation product of anthranilic acid (**18**). Using triphenylphosphine as a ligand, the mechanism of the hydroarylation of **19** becomes more complex (Scheme 3).

When triphenylphosphine is employed as a ligand in Heck reactions, a two-fold phosphine-coordinated palladium complex is known to be the catalytically active species. In the case of the hydroarylation of **19**, though, the yield of product **21** increases from 18 to 49% in parallel to an increase of the ligand to palladium ratio from 2:1 to 20:1 [20]. This surprising result can only be rationalized by proposing a rather stable palladium complex between an aromatic π-bond and the nitrogen lone pair. NMR spectroscopic investigations indicate that the nitrogen inversion is already fast at room temperature. A higher amount of phosphine ligand increases the ligand pressure on the palladium, thus destabilizing the proposed complex formation on the “wrong” inner double bond of the bicycle and giving the active palladium species a fair chance to approach the free olefinic side of the azatricyclic system. Two side products are formed, a formal nitrene-extrusion product **22** of the initial carbopalladation product of the pyridylpalladium iodide to **19** and the product of a selective reduction of the chloroiodopyridine **20** giving **23**. Additionally, the yield of **21** is strongly dependent on the nucleophilicity of the nitrogen: apparently, only electron-withdrawing groups such as the ester function of **19** prevent strong complexation and concomitant deactivitation of the palladium. While the parent system still gives the hydroarylation product in 28% yield, with an *N*-methyl-substituent the corresponding coupling product cannot be isolated even in traces. The argument of competitive *N*-complexation can also be applied when the unsuccessful reductive coupling of the pyridino-substituted analogue of **19** is discussed. The better stabilizing ligand triphenylarsine leads to the best yield of **21** (75%) [18,20].

**Scheme 4:**
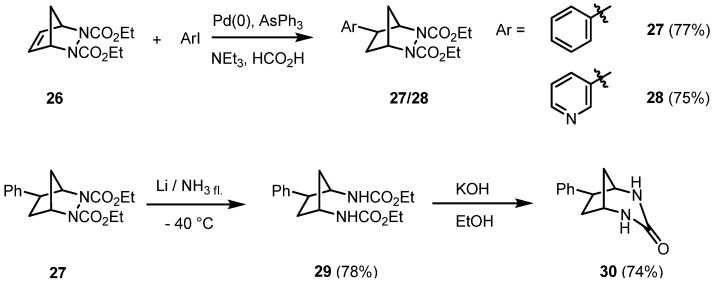
Hydroarylation of the 2,3-diazabicyclo[2.2.1]heptene **26**: stereoselective synthesis of intermediates.

At that point we became interested in the scope and limitations of reductive Heck reactions of (bishetero)bicyclic alkenes. 2,3-Diazabicyclic alkenes are of special interest as the N-N and C-N bond represent an internal point of cleavage. The diazabicyclic alkene **26** is easily accessible by a Diels-Alder reaction of cyclopentadiene with diethyl azodicarboxylate (DEAD). Its reductive C-C coupling reaction with (het)aryl halides in the presence of an *in situ* generated palladium catalyst, stabilized by triphenylarsine, afforded exclusively the *exo*-hydroarylation products **27** and **28** in good yields [21] (Scheme 4).

Again, it is feasible to substitute the formate in a hydroorganylation reaction by the nucleophilic phenylacetylene as shown in Scheme 2, resulting in a sequential C-C coupling reaction of **26**. The reductive cleavage of the N-N bond yielded the *cis*-1,3-diaminocyclopentane derivative **29** with an aryl substituent being *trans*-oriented. Treatment of the bisurethane **29** with a base afforded the cyclic urea **30** in a good yield, as shown in Scheme 4. 

**Scheme 5:**
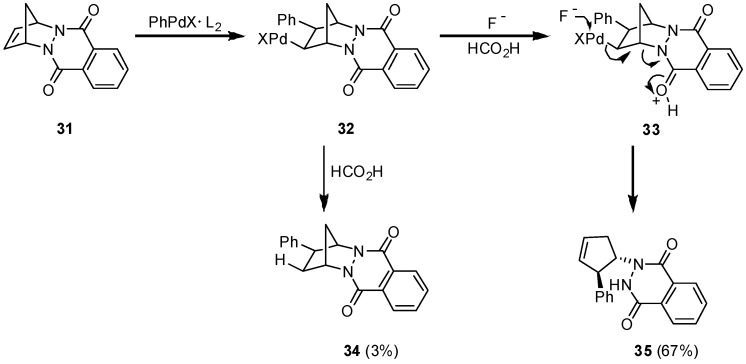
Competition in stereoselectivity: hydroarylation *vs*. hydrazidoarylation.

Encouraged by this result we tried the hydroarylation of the sterically more encumbered and more rigid tetracyclic Diels-Alder adduct **31** of 1,3-cyclopentadiene with the highly reactive azodienophile 1,4-phthalazinedione [22]. The reaction of **31** with iodobenzene in the presence of triphenylarsine as a ligand and additionally sodium fluoride at room temperature led to 3% of the expected hydroarylation product **34**, only, while 67% of **35** was formed as the product of a C-N cleavage reaction. Formally, the formation of **35** is the result of a 1,2-hydrazidoarylation on the primarily employed 1,3-cyclo-pentadiene (Scheme 5).

**Scheme 6:**
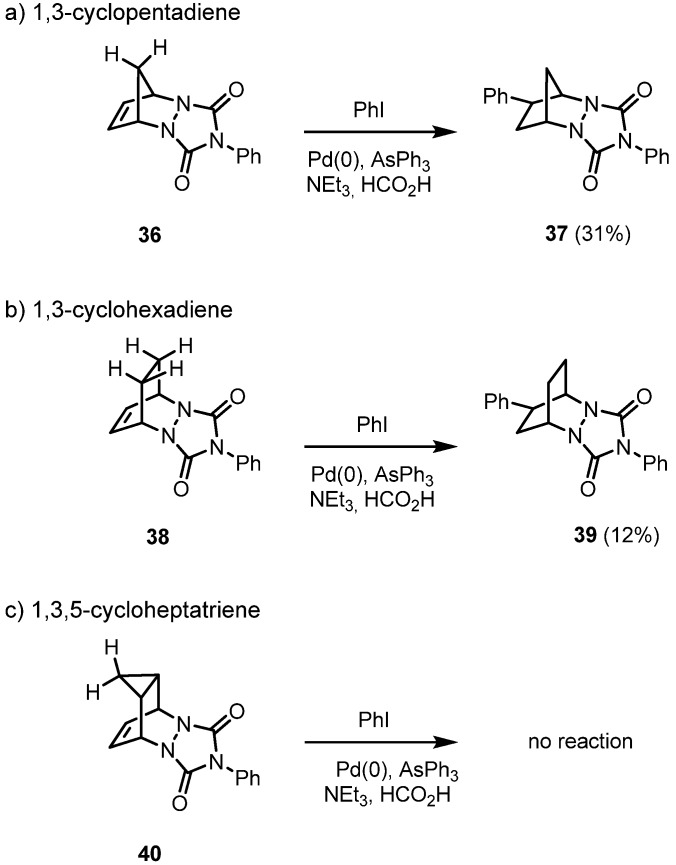
Hydroarylation of the cycloadducts of PTAD to cyclic dienes: dependence on the ring size.

The yield of the hydroarylation products under standard conditions is highly dependent on the rigid tricyclic system used: the PTAD cycloadduct of 1,3-cyclopentadiene **36** is best suited, and the yield drops by a factor of almost three in the case of the diazabicyclo[2.2.2]octene system **38**, due to both the lower reactivity of the less strained double bond and the steric interactions with both CH-groups. When the C=C double bond is covered by a cyclopropane ring as in compound **40**, *exo*-attack is no longer feasible (Scheme 6).

**Scheme 7:**
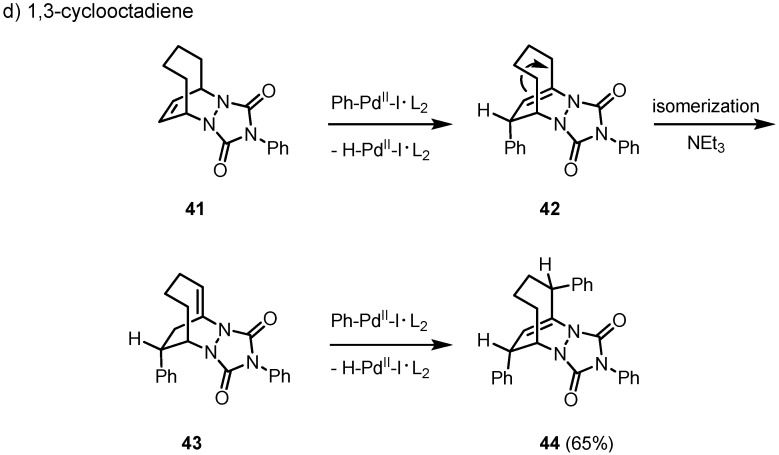
Mechanism of the hydroarylation of the PTAD cycloadduct of 1,3-cyclo-octadiene: ring flexibility leads to a twofold Heck reaction.

The key diazabicyclic systems become more flexible when a larger cycle is used, e.g. 1,3-cyclo-octadiene (1,3-COD). As an exception, in case of the PTAD cycloadduct **41** a twofold Heck reaction takes place in good yield to give **44** with an intermediate isomerization of the remaining *anti*-Bredt double bond from **42** to **43**. The structure of **44** was confirmed by an X-ray crystal structure analysis. Even under reductive conditions no hydroarylation product could be isolated. Therefore, in general the reductive elimination of hydridopalladium iodide to give **42** is apparently much faster than the reductive cleavage of the primarily formed carbopalladation product in the course of a hydroarylation reaction (Scheme 7).

**Scheme 8:**
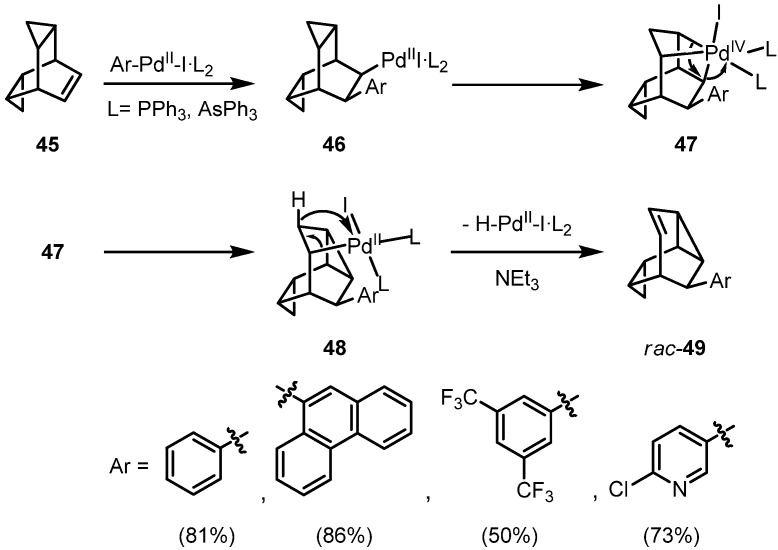
Mechanism of the palladium-catalyzed rearrangement of **45** under arylation.

Inter- or intramolecular sequential insertion reactions of a carbopalladation product into a strained cyclopropane C-C σ-bond were unknown for a long time. The easily accessible *endo*,*exo*-bishomobarrelene **44** represents a *cis*-allylcyclopropane model compound which can be attacked from one side only, due to the shielding by the second cyclopropane ring (Scheme 8).

Treatment of **45** with iodobenzene under standard hydroarylation conditions surprisingly leads to the formation of 9-phenylmonohomosemibullvalene (*rac***-49**) in 81% yield; not even traces of a hydroarylation product was formed. In comparison, reaction of the less strained cyclobuta-annelated bicyclo[2.2.2]hexene again results in a reductive arylation. The palladium-catalyzed phenylation of an allylcyclopropane unit under sequential rearrangement is novel and may be classified as the first example of a π,σ domino-Heck reaction. We suggest the pathway outlined in Scheme 8: after an initial *syn*-addition of the Ph-Pd-I species to the C=C double bond, the palladium(II) substituent can interact with the Walsh-orbitals of the neighboring *endo*-cyclopropane ring, leading by an oxidative addition from palladium(II) to palladium(IV). This highly strained octahedral complex can be stabilized by a reductive elimination to the σ-alkylpalladium(II) complex **48** under concomitant rearrangement. Compound **48** has the structural prerequisites for a final *syn*-elimination of hydridopalladium iodide with formation of racemic **49**. Addition of a chiral bisphosphine ligand such as (*R*)-BINAP allows one to control seven chiral centers within a single operation [23].

## 3. Conclusions

The palladium-catalyzed hydroarylation of bi- and oligocyclic systems has been proven a very valuable method for the stereoselective synthesis of the corresponding, frequently bioactive *exo*-arylated products. In the presence of chiral ligands, asymmetric induction of three centers appears feasible. The hydroarylation can be extended to *N*- and *O*-analogues. Electron-deficient palladium complexes allow C-N cleavage reactions, formally creating a two-step hydrazidoarylation of the corresponding cyclic 1,3-dienes. The efficiency of the hydroarylation correlates with the ring size of the starting material: rigid bicyclo[2.2.1]heptene systems are suited best; more flexible, larger bicycles even allow twofold Heck reactions, already. Under reductive conditions the presence of a neighboring cyclopropane ring can lead to a stereoselective π,σ domino-Heck reaction which proceeds under rearrangement and arylation, creating up to seven asymmetric centers in a single operation.
